# Phenotypic heterogeneity in familial epilepsies is influenced by polygenic risk for generalized and focal epilepsies

**DOI:** 10.1111/epi.18348

**Published:** 2025-03-06

**Authors:** Colin A. Ellis, Ruth Ottman, Michael P. Epstein, Epi4K Consortium, Samuel F. Berkovic, Karen L. Oliver

**Affiliations:** ^1^ Department of Neurology University of Pennsylvania Perelman School of Medicine Philadelphia Pennsylvania USA; ^2^ Department of Neurology and Epidemiology, and the Gertrude H. Sergievsky Center Columbia University Irving Medical Center New York New York USA; ^3^ Division of Translational Epidemiology and Mental Health Equity New York State Psychiatric Institute New York New York USA; ^4^ Department of Human Genetics Emory University School of Medicine Atlanta Georgia USA; ^5^ Epilepsy Research Centre, Department of Medicine University of Melbourne, Austin Health Heidelberg Victoria Australia; ^6^ Population Health and Immunity Division The Walter and Eliza Hall Institute of Medical Research Parkville Victoria Australia; ^7^ Department of Medical Biology University of Melbourne Melbourne Victoria Australia

**Keywords:** familial epilepsy, focal epilepsy, genetic generalized epilepsy, phenotypic heterogeneity, polygenic risk scores

## Abstract

**Objective:**

Although previous research shows that generalized and focal epilepsies have at least some distinct genetic influences, it remains uncertain why some families manifest both types of epilepsy. We tested two hypotheses: (1) families with both generalized and focal epilepsy carry separate risk alleles for both types; and (2) within mixed families, the type of epilepsy each individual manifests is influenced by the relative burden of separate risk alleles for generalized epilepsies and focal epilepsies.

**Methods:**

The Epi4K cohort included 711 individuals with epilepsy from 257 families (113 generalized families, 66 focal families, 78 mixed families). We calculated polygenic risk scores (PRSs) for genetic generalized epilepsy (GGE_PRS) and for focal epilepsy (Focal_PRS). We used mixed‐effects models to compare these PRSs between and within families, accounting for relatedness.

**Results:**

Compared to population controls, individuals in generalized families had elevated GGE_PRS (*p* < .001) but not elevated Focal_PRS (*p* = .50); focal family individuals had elevated Focal_PRS (*p* = .008) but not elevated GGE_PRS (*p* = .22); and individuals in mixed families had both elevated GGE_PRS and elevated Focal_PRS (both *p* < .001). Within mixed families, GGE_PRS was higher in individuals with generalized epilepsy than in individuals with focal epilepsy (*p* < .001), whereas we did not detect a difference in Focal_PRS between individuals with generalized and focal epilepsy (*p* = .46). The GGE_PRS value explained 10% of the variance in phenotype within mixed families.

**Significance:**

The occurrence of families with both generalized and focal epilepsy in separate individuals is explained at least partly by the chance co‐occurrence of distinct genetic risk alleles for generalized and focal epilepsies. Within mixed families, an individual's epilepsy type can be explained at least in part by the relative burden of risk alleles for genetic generalized epilepsy.

**Key points**

- We calculated polygenic risk scores for genetic generalized epilepsy (GGE_PRS) and for focal epilepsy (Focal_PRS) in individuals from 257 multiplex epilepsy families.
- Families with both generalized and focal epilepsies were partly explained by the chance co‐occurrence of distinct genetic risk alleles.
- Phenotypic heterogeneity within families was partly explained by the relative burden of risk alleles for genetic generalized epilepsy.
- These findings indicate an important role for common variants in explaining the heterogeneity between and within familial epilepsies.

## 1 INTRODUCTION

It is now well established that generalized and focal epilepsies have at least some distinct genetic influences. Studies of genetic epidemiology have long observed that twins and other family members tend to have concordant epilepsy types, suggesting the presence of distinct genetic contributions.^1^, ^2^, ^3^, ^4^ In the molecular genomics era, genome‐wide association studies (GWASs) of epilepsy have identified separate sets of common variant risk alleles for the different epilepsy types,^5^ and polygenic risk scores (PRSs) derived from those common variants have confirmed the dissociable roles of those distinct risk alleles in different types of epilepsy.^6^, ^7^

**FIGURE 1:**
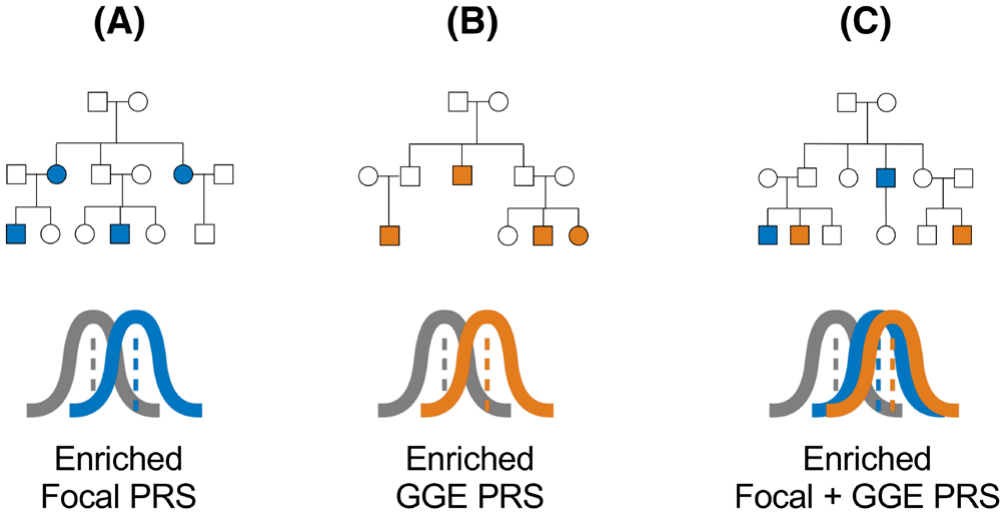
Hypothesis about genetic mechanism of mixed families. Focal epilepsies (A) and generalized epilepsies (B) are known to have at least partially distinct genetic determinants. Mixed families (C) that manifest both types of epilepsy are hypothesized to be explained at least in part by chance co‐occurrence of distinct genetic determinants for focal and generalized epilepsies. Colored symbols in pedigrees represent diagnosed phenotypes (blue = focal epilepsy, orange = generalized epilepsy). The diagrams beneath the pedigrees represent hypothetical normal distributions of polygenic risk scores for individuals from hypothetical families as represented above (blue = PRS for focal epilepsy, orange = PRS for genetic generalized epilepsy, gray = population controls). Figure adapted from reference^9^.

What is less clear is why family members, who share common genetics, sometimes manifest different types of epilepsy. These mixed families have historically been interpreted as evidence of shared genetic mechanisms underlying different epilepsy types,^2^, ^8^ with individual phenotypes presumably determined by nongenetic factors. An alternative hypothesis (Figure 1) is that distinct genetic determinants of focal and generalized epilepsy may co‐occur by chance in these mixed families, with individual phenotypes determined by the relative balance of different low‐effect‐size risk alleles that an individual happens to inherit.

In a recent study analyzing familial aggregation of different epilepsy types, we found clinical evidence to support this hypothesis. Within mixed families, we found that pairs of relatives with the same epilepsy types (concordant pairs) were more closely related than pairs of relatives with different epilepsy types (discordant pairs).^9^ This suggested that heterogeneity within mixed families can be explained at least in part by the chance co‐occurrence of distinct genetic determinants of focal and generalized epilepsy.

Here, we tested this hypothesis using molecular genetic data. PRSs provide individual‐level estimates of genetic risk based on the aggregate effect of common genetic variants across the genome. We used the most recent GWAS of epilepsy (International League Against Epilepsy [ILAE] 2023)^5^ to calculate separate PRSs for genetic generalized epilepsies (GGEs) and for focal epilepsies. We calculated these PRSs in a cohort of familial epilepsies collected by the Epi4K Consortium.^10^, ^11^ These families contained three or more affected individuals with epilepsy of unknown cause.

We tested two hypotheses. First, we sought to understand heterogeneity between families. We compared individuals from the mixed families to those from homogeneous families containing only genetic generalized epilepsies, or only focal epilepsies. This analysis directly tested the hypothesis that mixed families are explained in part by the chance co‐occurrence of separate risk alleles for generalized epilepsies and focal epilepsies. Second, we sought to understand heterogeneity within families. We tested the hypothesis that individual phenotypes within mixed families are determined in part by each individual's relative burden of risk alleles for genetic generalized epilepsies and focal epilepsies.

## 2 METHODS

### 2.1 Ethics statement

The Epi4K study was approved by research ethics committees at each participating site. All participants provided written informed consent to participate. This analysis used de‐identified data from that study and did not require additional ethics approval. The QSkin Sun and Health Study was approved by QIMR Berghofer Medical Research Institute's Human Research Ethics Committee (P1309, P2034), including amendments to share data with this study.

### 2.2 Data collection

The Epi4K multiplex families cohort has been described in detail elsewhere.^10^ Briefly, each family contained three or more individuals with epilepsy of unknown cause. Individuals with known acquired causes, structural brain lesions, and moderate or severe intellectual disability were excluded. Ascertainment occurred at multiple centers in the United States, Canada, Israel, Wales, Ireland, Australia, and New Zealand.

Individuals were classified by expert clinicians into epilepsy types based on a review of all available clinical data including seizure semiology, electroencephalography (EEG), and neuroimaging. The following epilepsy types were included in this analysis: nonacquired focal epilepsy, GGEs, combined focal and generalized epilepsy, and unclassified epilepsy. Combined epilepsy was diagnosed only when there was clear clinical and/or EEG evidence of both generalized and focal epilepsy in the individual.^9^, ^10^ Unclassified epilepsy was assigned when cases had only tonic–clonic seizures or lacked clear semiology and did not have epileptiform EEG.

Based on the classifications of individuals, families were then classified into the following categories: generalized families contained only individuals with generalized or unclassified epilepsy; focal families contained only individuals with focal or unclassified epilepsy; and mixed families contained generalized and focal epilepsy in separate individuals, or any individuals with combined epilepsy.

Families with known causal pathogenic variants were excluded from the original Epi4K families cohort. Since the cohort was first assembled, five families had pathogenic variants identified, and nine families have copy number variants associated with epilepsy.^12^ In many cases these families had unaffected variant carriers and/or affected non‐carriers, meaning that the variants were not complete explanations for the familial epilepsies. We chose to include these families because polygenic risk may be relevant even in families with putative pathogenic variants.^13^, ^14^

Population controls were obtained from the QSkin Sun and Health Study, a prospective cohort study of individuals randomly sampled from the Australian state of Queensland.^15^

### 2.3 Genotyping and polygenic risk scores

These methods have been described in detail previously.^7^ Briefly, Epi4K samples were genotyped using Illumina HumanCore, Human Multi‐Ethnic, or Multi‐Ethnic Global Array. QSkin samples were genotyped using Illumina Global Screening Array. Following standard quality controls, we excluded single‐nucleotide polymorphisms (SNPs) with missingness above 2%, minor allele frequency below .5%, or Hardy–Weinberg equilibrium *p* < 10^−6^, and we excluded samples with heterozygosity rates above .2, missing genotypes above 2%, or failed sex check.^16^ We restricted the cohort to European ancestry based on principal component analysis of 1000 genomes data (Figure S1). This resulted in the exclusion of 24 non‐European families. Imputation to the HRC r1.1 2016 (GRCh37/hg19) reference panel was performed using Minimac4 on the Michigan Imputation Server with preimputation phasing using Eagle v2.4.^17^, ^18^ We included imputed SNPs with high‐quality scores *R* ^2^ > .9, and repeated the above quality controls before and after merging the datasets. Ancestry principal component analysis was performed using PCAir to account for relatedness.^19^ We generated pairwise identity‐by‐descent estimates to identify and remove duplicate samples, and to confirm that genetic relatedness matched expected pedigree relationships within families.

We calculated PRSs for each sample using summary statistics from the largest available epilepsy GWAS (European‐only ancestry sample).^5^ To avoid potential PRS bias, we excluded 26 Epi4K families, where sample manifest records confirmed that members contributed to the source GWAS^20^ through an earlier Epi4K project arm.^21^ We calculated two separate PRSs, one for genetic generalized epilepsy (GGE_PRS) and one for focal epilepsy (Focal_PRS), using PRSice‐2.^22^ We applied a significance threshold of *p* < .5 to select variants for inclusion in the PRS models, as this was the optimal threshold determined by previous epilepsy PRS studies.^6^, ^7^ As a sensitivity analysis we used more stringent thresholds of *p* < .01 and *p* < .00001. As a negative control experiment we calculated PRSs for asthma and irritable bowel syndrome based on publicly available summary statistics from an independent GWAS,^23^ also thresholded at *p* < .5. All PRS values were normalized to a standard normal distribution with a mean of 0 and standard deviation (SD) of 1.

### 2.4 Statistical analysis

#### 2.4.1 Heterogeneity between families

First, we tested the hypothesis that mixed families are explained in part by the chance co‐occurrence of separate risk alleles for generalized epilepsies and focal epilepsies. We compared the GGE_PRS and Focal_PRS of affected members of generalized families, focal families, mixed families, and population controls. We used linear mixed‐effects models that treated each PRS as outcome, family type as the predictor of interest with controls as the reference category, four principal components as fixed‐effects covariates to account for population substructure, and the genetic relatedness matrix as random‐effects covariate to account for relatedness within families. This approach gives more weight to more closely related individuals, accounting for the influence of family structure and the fact that closer relatives share more genetic variants in common than distant relatives. The first four principal components collectively explained 95% of the total genetic variance in the study cohort (Figure S2). For each model, we compared each family type to controls.

As a sensitivity analysis, we repeated the analysis after removing individuals with unclassified epilepsy. As negative control experiments to assess for any systematic bias between cases and controls (e.g., due to differences in ancestry or genotyping platforms), we repeated the same analysis using PRS scores for the non‐neurologic conditions asthma and inflammatory bowel disease, expecting no differences between cases and controls. We selected these two conditions as negative controls because they are non‐neurologic conditions without known genetic overlap with epilepsy, and we chose two conditions in order to demonstrate the reproducibility of the negative control.

#### 2.4.2 Heterogeneity within mixed families

These analyses tested the hypothesis that individual phenotypes within mixed families are determined in part by the relative burden of separate risk alleles for generalized epilepsies and focal epilepsies. These analyses were restricted to mixed families and their member individuals with only generalized epilepsy or only focal epilepsy.

First, we used linear mixed‐effects models to compare the GGE_PRS and Focal_PRS of individuals with generalized epilepsies compared to individuals with focal epilepsies, again adjusting for the first four ancestry components as fixed‐effects covariates and the genetic relatedness matrix as random‐effects covariate.

Next we measured the extent to which the combination of GGE_PRS and Focal_PRS were predictive of an individual's phenotype within these mixed families. We constructed a logistic mixed‐effects logistic regression model using phenotype (generalized or focal) as the outcome variable; GGE_PRS, Focal_PRS, and their interaction as fixed effects; and family identifier as the random effect. Odds ratios in logistic mixed‐effects models represent the effect of PRS on phenotype holding all other covariates constant including the random effect of family, that is, the within‐family (conditional) effect of PRS. We estimated the total variance explained by the model using Tjur's coefficient of determination, a pseudo‐*R* ^2^ estimate for mixed‐effects models.^24^ We also constructed subsets of the full model containing only GGE_PRS, only Focal_PRS, and GGE_PRS plus Focal_PRS without their interaction.

We also performed pairwise analysis of the difference in PRS between pairs of relatives within mixed families. Whereas the mixed‐effects models described above adjusted for relatedness as a covariate, this approach directly compares individuals only to their own family members. Because related individuals share a proportion of their DNA, this approach has the benefit of a lower risk of bias due to population stratification, at the expense of lower power to detect true signal. (See Methods S1 for additional details).

Analyses were performed in R version 4.4.1.

## 3 RESULTS

### 3.1 Cohort characteristics

The cohort consisted of 711 individuals with epilepsy from 257 families. Classifications of individuals and families are shown in Table 1, as described in previous studies.^9^, ^10^ The cohort was 58% female. The number of sampled individuals per family in the cohort overall was median 3, range 2–6, with no significant difference between family types (Kruskal–Wallis test, *p* = .37). Although every family contained three or more individuals with epilepsy, not every individual had available DNA that passed quality controls. The control cohort was 15 921 unrelated individuals, of whom 55% were female.

**TABLE 1:** Cohort of familial epilepsies.

	Family classification			Total
	Generalized	Focal	Mixed	
Families	113	66	78	257
Individuals	309	158	244	711
Individuals per family, median (range)	3 (2–6)	3 (2–5)	3 (2–6)	3 (2–6)
Epilepsy types				
GGE	264	0	100	364
Focal	0	140	84	224
Combined	0	0	29	29
Unclassified	45	18	31	94

*Note*: The cohort was derived from previous studies of the Epi4K cohort,^9^, ^10^ limited here to participants of European ancestry with DNA that passed quality controls.

### 3.2 Heterogeneity between families: Mixed families have risk alleles for both GGE and focal epilepsy

**FIGURE 2:**
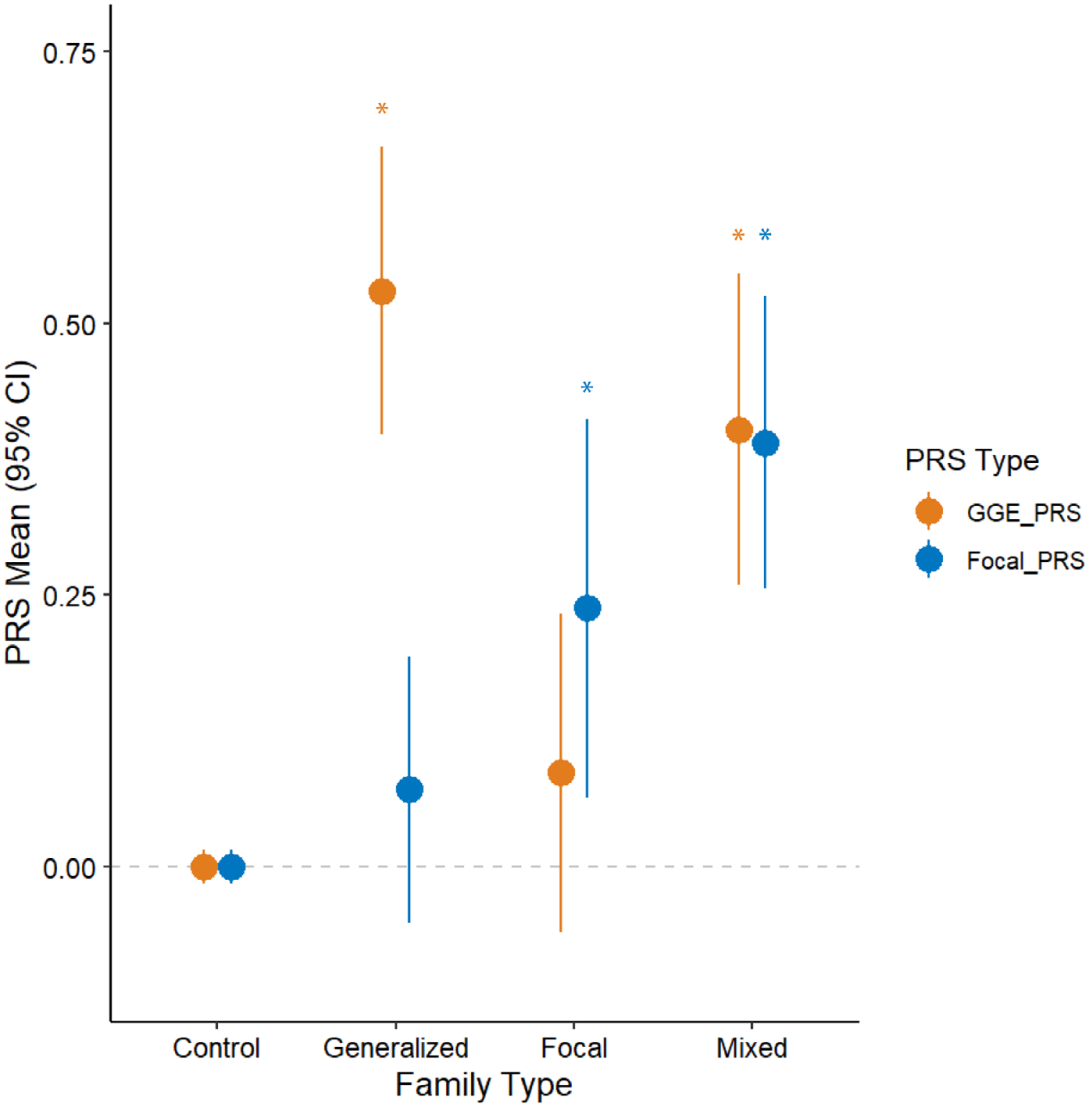
Mixed families have risk alleles for both generalized and focal epilepsy. Shown are the mean (95% CI) PRS for GGE epilepsy (orange) and focal epilepsy (blue) for members of generalized families (*n* = 309), focal families (*n* = 158), mixed families (*n* = 244), and controls (*n* = 15 921). Stars indicate significant increase in PRS compared to controls (*p* < .01). Abbreviations: CI = confidence interval, PRS = polygenic risk score, GGE = genetic generalized epilepsy.

This analysis tested the hypothesis that the occurrence of mixed families is explained in part by the chance co‐occurrence of separate risk alleles for generalized epilepsies and focal epilepsies. Results are shown in Figure 2 and Table 2. Generalized families had elevated GGE_PRS (*p* < .001) but not elevated Focal_PRS (*p* = .50); focal families had elevated Focal_PRS (*p* = .008) but not elevated GGE_PRS (*p* = .22); and mixed families had both elevated GGE_PRS and elevated Focal_PRS (both *p* < .001). Sensitivity analysis showed similar results after removing unclassified individuals (Figure S3). In negative control experiments, all family types had PRSs similar to controls for asthma and inflammatory bowel disease (Figure S4).

**TABLE 2:** PRS in families, by family classification.

Family type	PRS for generalized epilepsy		PRS for focal epilepsy	
	Coefficient (SE)	*p*‐value	Coefficient (SE)	*p*‐value
Generalized	.51 (.08)	<.001	.05 (.07)	.50
Focal	.12 (.10)	.22	.26 (.10)	.008
Mixed	.34 (.08)	<.001	.28 (.08)	<.001

*Note*: Coefficients and standard error (SE) values are from linear mixed‐effects models with PRS as outcome, family type as the predictor of interest with controls as the reference category, four ancestry components as fixed‐effects covariates, and the genetic relatedness matrix as random‐effects covariate to account for relatedness within families. Abbreviations: PRS = polygenic risk scores.

### 3.3 Heterogeneity within mixed families: GGE Risk alleles influence individual phenotype

**FIGURE 3:**
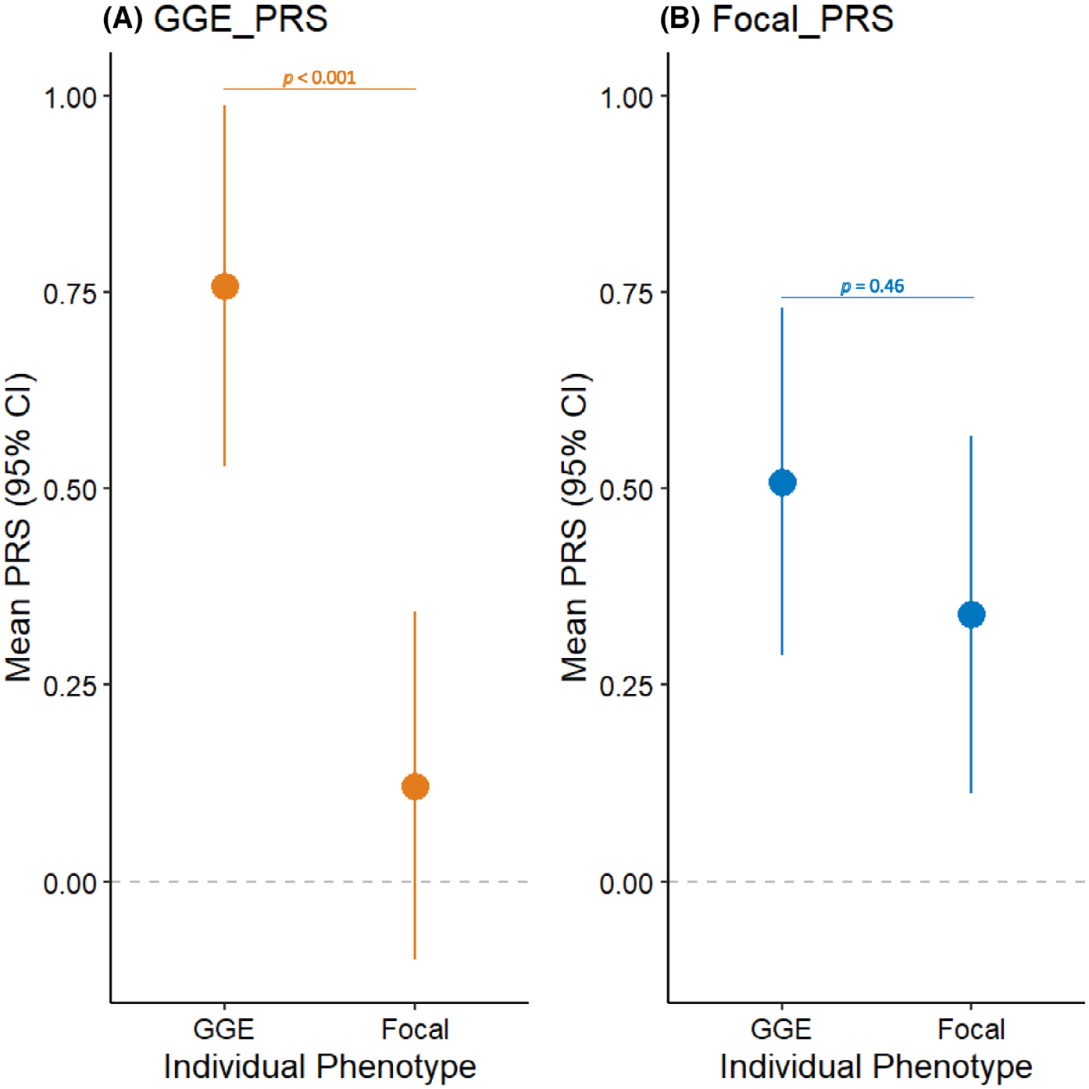
Heterogeneity within mixed families: Cohort analysis. Shown are the mean (95% CI) PRS for GGE epilepsy (orange/panel A) and focal epilepsy (blue/panel B) for members of mixed families who have generalized epilepsy (*n* = 100) or focal epilepsy (*n* = 84).

This analysis was restricted to the 78 mixed families and their individual members with generalized epilepsy (*n* = 100) or focal epilepsy (*n* = 84). As shown in Figure 3, GGE_PRS was higher in individuals with generalized epilepsy compared to individuals with focal epilepsy (coefficient .48, standard error [SE] .15, *p* = .001). In contrast, Focal_PRS did not differ between individuals with generalized epilepsy and individuals with focal epilepsy (coefficient .02, SE .15, *p* = .90).

Next we measured the extent to which PRS for generalized and focal epilepsies predicted an individual's phenotype within these mixed families. In the mixed‐effects logistic regression model that included GGE_PRS, Focal_PRS, and their interaction, only GGE_PRS was a significant predictor of phenotype (Table 3). The full model explained 10.8% of the variance in phenotype (Tjur's coefficient of determination = .108). This was driven largely by GGE_PRS, a partial model containing only the GGE_PRS predictor explained 10.0% of the variance in phenotype, whereas a partial model containing only the Focal_PRS predictor explained only .8% of the variance in phenotype (Table S2).

**TABLE 3:** Polygenic risk scores for generalized and focal epilepsy as predictors of individual phenotypes within mixed families.

Predictor	Coefficient	SE	OR (95% CI)	*p*‐value
GGE_PRS	.57	.16	1.77 (1.28, 2.45)	<.001
Focal_PRS	−.17	.18	.84 (.59, 1.19)	.33
GGE_PRS*Focal_PRS	.08	.12	1.08 (.85, 1.38)	.52

*Note*: Coefficients and standard error (SE) values are from logistic mixed‐effects logistic regression model using individual phenotype (generalized or focal) as outcome variable; GGE_PRS, Focal_PRS, and their interaction as fixed effects; and family as random effect. Odds ratios in logistic mixed effects models represent the effect of PRS on phenotype holding all other covariates constant including the random effect of family, i.e., the within‐family (conditional) effect of PRS. Abbreviations: CI = confidence interval, GGE = genetic generalized epilepsy, OR = odds ratio, PRS = polygenic risk scores, SE = standard error.

Repeating the analysis using more stringent *p*‐value thresholds for selecting the variants included in the PRS models reduced the difference of GGE_PRS between individuals with generalized and individuals with focal epilepsy. There remained no difference of PRS_Focal between individuals with generalized and individuals with focal epilepsy (Figure S5).

Pairwise analysis within families showed findings similar to those of the primary analysis (Figure S3, Table S1). Pairwise difference in GGE_PRS was higher in individuals with generalized epilepsy than their relatives with focal epilepsy. Pairwise difference in Focal_PRS was similar for individuals with focal epilepsy and their relatives with generalized epilepsy.

## 4 DISCUSSION

This study had two key findings. The first was that family‐level classification into generalized, focal, and mixed families was associated, respectively, with an increased burden of risk alleles for genetic generalized epilepsy alone, an increased risk alleles for focal epilepsy alone, and an elevated PRS for both. This provides molecular genetic evidence to support the hypothesis that mixed families occur at least in part due to the chance co‐occurrence of the different sets of common genetic risk variants for genetic generalized epilepsy and focal epilepsy. As a simplified conceptual model, families with a high burden of risk alleles for generalized epilepsy manifest generalized epilepsies; families with risk alleles for focal epilepsy manifest focal epilepsies; and families that inherit both types of risk alleles can manifest both types of epilepsy.

The second key finding was that, within mixed families, the type of epilepsy manifested by an individual was strongly influenced by risk alleles for genetic generalized epilepsy (GGE_PRS). In contrast, although the burden of risk alleles for focal epilepsy (Focal_PRS) was elevated in these individuals, it did not help differentiate their individual phenotypes. Conceptually, this suggests that when a family inherits risk alleles for both types of epilepsy, the relative burden of GGE_PRS acts as “kingmaker” with regard to which type of epilepsy an individual manifests. This is consistent with the observation that generalized epilepsy is more strongly polygenic than focal epilepsy in the general population, based on evidence from both epidemiology^1^, ^25^, ^26^, ^27^ and GWASs.^5^ Our findings extend the same logic to heterogeneity within families.

Phenotypic heterogeneity is a common but incompletely understood phenomenon in familial epilepsies. This observation is often discussed in the context of families that carry a highly penetrant pathogenic variant (“monogenic”).^28^ For example, families with pathogenic *SCN1A* variants can have heterogeneous phenotypes ranging from severe epileptic encephalopathy to mild febrile seizures, and various seizure types including both generalized and focal.^29^, ^30^ Mouse models of heterozygous *Scn1a* knockout can reproduce Dravet syndrome in one mouse strain but cause no overt phenotype in a different mouse strain.^31^ These observations suggest that an individual's genetic liability for epilepsy, now measurable through PRSs, can influence the phenotypic expression of epilepsy. In the current study we focused on common familial epilepsies with non‐Mendelian inheritance, which represent the majority of familial epilepsies. The role of polygenic risk from common variant risk alleles in such families is likely to be especially relevant.

Risk alleles for genetic generalized epilepsy and focal epilepsy are not mutually exclusive. Analysis of GWAS data has shown correlations between the risk alleles for different epilepsy types.^5^ This correlation would make it harder to detect differences in PRSs between different epilepsy types and different family types, biasing toward the null. If the correlation was perfect, GGE_PRS and Focal_PRS would be measuring the same thing, and would not show different patterns within individuals or families. Our findings suggest that, to the extent that GGE and focal epilepsy have different risk alleles (or different weights for shared risk alleles), these different risk profiles contribute meaningfully to the phenotypic heterogeneity of familial epilepsies.

In a previous study, we analyzed the pedigrees of Epi4K families for familial aggregation of epilepsy phenotypes.^9^ That study found that, in mixed families, pairs of relatives with concordant epilepsy types were more closely related than discordant pairs, which we hypothesized was due to chance co‐occurrence of the separable genetic determinants of focal and generalized epilepsies. Here we tested that hypothesis more directly using molecular genetic data. Our findings provide additional and more direct support for the hypothesis, by demonstrating that the common variants identified by GWASs are able to explain part of the observed heterogeneity between families and within families. We accounted for genetic relatedness as a covariate, which is important because closer relatives naturally share more genetic variants in common and will have more similar PRSs by chance. That is, our findings of different patterns of GGE_PRS and Focal_PRS between different family types, and between different individual phenotypes within mixed families, are present when comparing individuals of similar relatedness. These findings also complement a prior study showing that the burden of risk alleles for all types of epilepsy differs between affected and unaffected individuals within families.^7^ Future studies could explore the transmission of polygenic risk from generation to generation within families by extending the concept of the polygenic transmission disquilibrium test to multiplex families.^32^

Our findings that generalized families had elevated GGE_PRS but not Focal_PRS, whereas focal families had elevated Focal_PRS but not GGE_PRS (Figure 2), contrast with the findings of Leu and colleagues,^6^ who found that both PRS subtypes were elevated in individuals with both types of epilepsy. The two studies differed in several ways that may explain this contrast. Our study used a larger GWAS to calculate PRS, possibly increasing the accuracy of the risk alleles and increasing the discriminatory power of the two types of PRSs. Our study focused on familial epilepsies, whereas the previous study included unselected epilepsy cases. When families contain multiple individuals with the same type of epilepsy, they may be particularly enriched in risk alleles for that epilepsy type, or particularly depleted in risk alleles for other epilepsy types, compared to the unselected population of all individuals with epilepsy. This might also explain why our mixed families, perhaps more like the unselected population, showed more complex PRS profiles, with elevated Focal_PRS in individuals with GGE. Of interest, Leu et al. also found that GGE_PRS was higher in individuals with generalized epilepsy than individuals with focal epilepsy epilepsy, whereas Focal_PRS was not different between the two groups of individuals, which parallels our findings within the mixed families (Figure 3).

The cohort included individuals with unclassified epilepsy whom we included in family‐level analyses. These individuals presumably had either generalized or focal epilepsy (or combined epilepsy) and could have been classified had more information been available (for example, video‐EEG). Sensitivity analysis suggested that including these individuals did not bias the analysis of heterogeneity between different types of families. Because related individuals share the same epilepsy types more often than not,^1^, ^2^ many unclassified individuals in otherwise homogeneous families likely had the same epilepsy type as the rest of the family. To the extent that some unclassified individuals actually had epilepsy types that were discordant with the rest of the family, those families would resemble mixed families, making different PRS patterns in different family categories harder to detect.

Our study had several limitations. The study was restricted to people of European ancestry because that was the population of the GWAS used to derive the PRS and the Epi4K cohort contained too few non‐European families for meaningful analysis. Familial epilepsies are well suited to study genetic heterogeneity, but may not be representative of all epilepsies. We studied generalized and focal epilepsies, but within those broader categories there are different syndromes that may have their own specific genetics that this study was not powered to study. Misclassification of seizure types is a possible limitation, despite systematic phenoytping by expert clinicians to help minimize this risk. Families with mixed epilepsy types are relatively rare, and we did not have access to independent replication cohorts to validate our findings, which should be a goal of future research. Our cases and controls were genotyped on different platforms, although our negative control experiments suggest that this did not introduce systematic bias in the PRS calculations. We calculated PRSs using the method of significance thresholding similar to prior studies of epilepsy,^6^, ^7^ although there are alternative methods that use all available variants,^33^, ^34^ and we expect PRS calculation methods to continue evolving in the future.

## 5 CONCLUSION

Mixed families with generalized epilepsy and focal epilepsy in separate individuals are explained at least in part by the chance co‐occurrence of distinct genetic risk alleles for generalized epilepsy and focal epilepsy. Within mixed families, phenotypic heteroegenity can be explained at least in part by the relative burden of risk alleles for generalized epilepsy. These findings indicate an important role for common variants in explaining the heterogeneity between and within familial epilepsies, and advance our understanding of the genetic basis of epilepsy and its subtypes.

## AUTHOR CONTRIBUTIONS

C.A.E., R.O., S.F.B., and K.L.O. contributed to study concept and design. All authors contributed to data acquisition, data analysis, and drafting of the manuscript and figures.

## CONFLICT OF INTEREST STATEMENT

None of the authors has any conflict of interest to disclose.

## ETHICAL PUBLICATION STATEMENT

We confirm that we have read the Journal's position on issues involved in ethical publication and affirm that this report is consistent with those guidelines.

## Supporting information


Appendix S1.

Figure S1.

## Data Availability

The data that support the findings of this study are openly available in dbGaP at https://www.ncbi.nlm.nih.gov/gap/.
