# Supplementary material for: Phenotypic heterogeneity in familial epilepsies is influenced by polygenic risk for generalized and focal epilepsies
Source: Epilepsia. 2025 Mar 6;66(6):2036–45. doi: 10.1111/epi.18348 (PMC12169383; doi:10.1111/epi.18348)
Supplement: Supplementary file 1 — Appendix S1. [file EPI-66-2036-s002.docx]

**Phenotypic heterogeneity in familial epilepsies is influenced by polygenic risk for generalized and focal epilepsies**

Colin A. Ellis, Ruth Ottman, Michael P. Epstein, Epi4K Consortium, Samuel F. Berkovic, Karen L. Oliver

Supplementary Appendix: Consortia Contributors

| **Name** | **Affiliation** |
| --- | --- |
| Bassel Abou-Khalil | Department of Neurology, Vanderbilt University Medical Center, Nashville, Tennessee 37232 USA. |
| Zaid Afawi Afawi | Ben-Gurion University of the Negev, Beer-Sheva, Israel and  Erasmus MC, Rotterdam, the Netherlands. |
| Andrew S. Allen | Center for Statistical Genetics and Genomics, Department of Biostatistics and Bioinformatics, Duke University, Durham, North Carolina 27710, USA. |
| Dina Amrom | Neurogenetics Unit, Montreal Neurological Institute and Hospital, Montreal, Quebec, Canada; Department of Neurology & Neurosurgery, McGill University, Montreal, Quebec, Canada |
| Eva Andermann | Neurogenetics Unit, Montreal Neurological Institute and Hospital, Montreal, Quebec, Canada; Epilepsy Research Group, Montreal Neurological Institute and Hospital, Montreal, Quebec, Canada; Department of Neurology & Neurosurgery, McGill University, Montreal, Quebec, Canada; Department of Human Genetics, McGill University, Montreal, Quebec, Canada. |
| Jocelyn F. Bautista | Department of Neurology, Cleveland Clinic Lerner College of Medicine, Epilepsy Center of the Cleveland Clinic Neurological Institute, Cleveland, Ohio 44195 USA. |
| Susannah T. Bellows | Epilepsy Research Centre, Department of Medicine, University of Melbourne (Austin Health), Heidelberg, Victoria 3084, Australia. |
| Samuel F. Berkovic | Epilepsy Research Centre, Department of Medicine, University of Melbourne, Austin Health, Heidelberg, Victoria, Australia. |
| Judith Bluvstein | Department of Neurology, Zucker Hofstra School of Medicine, Lenox-Hill Hospital, New York, NY 10075. |
| Alexis Boro | Department of Neurology, Montefiore Medical Center, Albert Einstein College of Medicine, Bronx, New York, 10467 USA. |
| Rosemary Burgess | Epilepsy Research Centre, Department of Medicine, University of Melbourne (Austin Health), Heidelberg, Victoria 3084, Australia. |
| Gregory D. Cascino | Division of Epilepsy, Mayo Clinic, Rochester, Minnesota 55905 USA. |
| Seo-Kyung Chung | Neurology and Molecular Neuroscience Research, Institute of Life Science, Swansea University Medical School, Swansea University, Swansea SA2 8PP, UK; Kids Neuroscience Centre, Kids Research, Children's Hospital at Westmead, Sydney, NSW 2145, Australia. |
| Damian Consalvo | Epilepsy Section, Neurology Division, Ramos Mejía Hospital, Buenos Aires, 1221, Argentina. |
| Patrick Cossette | Département de neurosciences, Université de Montréal, Centre Hospitalier de l’Université de Montréal, Quebec, Canada. |
| Douglas E. Crompton | Department of Neurology, Northern Health, Epping, Victoria 3076, Australia; Epilepsy Research Centre, Department of Medicine, University of Melbourne (Austin Health), Heidelberg, Victoria 3084, Australia. |
| Patricia Crumrine | Medical Epilepsy Program & EEG & Child Neurology, Children’s Hospital of Pittsburgh of UPMC, Pediatrics, University of Pittsburgh School of Medicine, Pittsburgh, Pennsylvania 15224 USA. |
| Sarah W. Curtis | Department of Human Genetics, Emory University School of Medicine, Atlanta, Georgia, 30322, USA. |
| Norman Delanty | Department of Neurology, Beaumont Hospital, and FutureNeuro Research Centre, Royal College of Surgeons in Ireland, Dublin 9, Ireland. |
| Orrin Devinsky | NYU Comprehensive Epilepsy Center, New York University, Department of Neurology, NYU School of Medicine, New York, New York 10016, USA. |
| Dennis Dlugos | Department of Neurology and Pediatrics, The Children’s Hospital of Philadelphia, Perelman School of Medicine at the University of Pennsylvania, Philadelphia, Pennsylvania 19104, USA. |
| Colin A. Ellis | Department of Neurology, Perelman School of Medicine at the University of Pennsylvania, Philadelphia, Pennsylvania 19104, USA. |
| Michael P. Epstein | Department of Human Genetics, Emory University School of Medicine, Atlanta, Georgia, 30322, USA. |
| Miguel Fiol | Department of Neurology, Epilepsy Care Center, University of Minnesota Medical School, Minneapolis 55414 USA. |
| Nathan B. Fountain | FE Dreifuss Comprehensive Epilepsy Program, University of Virginia, Charlottesville, Virginia 22908 USA. |
| Catharine Freyer | Department of Neurology, University of California, San Francisco, San Francisco, California 94143 USA. |
| Eric B. Geller | Institute of Neurology and Neurosurgery at Cooperman Barnabas Medical Center , Saint Barnabas Medical Center, Livingston NJ, USA. |
| Tracy Glauser | Division of Neurology, Cincinnati Children's Hospital Medical Center, Cincinnati, Ohio 45229 USA. |
| Simon Glynn | Department of Neurology and Neuroscience Graduate Program, University of Michigan Medical Center, Ann Arbor, and Ann Arbor Veterans Administration Healthcare System, Ann Arbor, Michigan, USA. |
| David B. Goldstein | Institute for Genomic Medicine, Columbia University Medical Center, New York, New York 10032 USA. |
| Micheline Gravel | Centre of Excellence in Neuromics and CHUM Research Center, Université de Montréal, Quebec, H2X 0A9, Canada |
| Kevin Haas | Department of Neurology, Vanderbilt University Medical Center, Nashville, Tennessee 37232 USA. |
| Rebekah V. Harris | Epilepsy Research Centre, Department of Medicine, University of Melbourne (Austin Health), Heidelberg, Victoria 3084, Australia. |
| Sheryl Haut | Department of Neurology, Montefiore Einstein, 111 East 210th St, Bronx, NY 1046. |
| Erin L. Heinzen | Division of Pharmacotherapy and Experimental Therapeutics, Eshelman School of Pharmacy, and Department of Genetics, School of Medicine, University of North Carolina, Chapel Hill, North Carolina, USA. |
| Olivia J. Henry | Department of Molecular Medicine and Surgery, Karolinksa Institutet, Stockholm 17177 , Sweden. |
| Sucheta Joshi | Department of Pediatrics , University of Michigan, Ann Arbor, Michigan 48109 USA. |
| Heidi E. Kirsch | Department of Neurology, University of California, San Francisco, San Francisco, California 94143 USA. |
| Sara Kivity | Epilepsy Unit, Schneider Children’s Medical Center of Israel, Petach Tikvah, Israel. |
| Robert C. Knowlton | Department of Neurology, University of California, San Francisco, San Francisco, California 94143 USA. |
| Eric Kossoff | Department of Neurology, Johns Hopkins Hospital, Baltimore, Maryland 21287 USA. |
| Ruben Kuzniecky | Department of Neurology, Zucker Hofstra School of Medicine, Lenox-Hill Hospital, New York, NY 10075. |
| Rebecca Loeb | Division of Gastroenterology and Hepatology, University of California, San Francisco, San Francisco, California 94143 USA. |
| Daniel H. Lowenstein | Department of Neurology, University of California, San Francisco, San Francisco, California 94143 USA. |
| Anthony G. Marson | Department of Pharmacology and Therapeutics, University of Liverpool, Clinical Sciences Centre, Lower Lane, Liverpool L9 7LJ, UK |
| Mark McCormack | Molecular & Cellular Therapeutics, Royal College of Surgeons in Ireland, Dublin 2, Ireland; and Department of Genetics, Universitair Medisch Centrum Utrecht, Utrecht, the Netherlands. |
| Shannon M. McGuire | Clinical Neurology, Children’s Hospital Epilepsy Center of New Orleans, New Orleans, Louisiana 70118 USA. |
| Kevin McKenna | Department of Neurology, University of California, San Francisco, San Francisco, California 94143 USA. |
| Heather C. Mefford | Center for Pediatric Neurological Disease Research, St. Jude Children's Research Hospital, Memphis, TN, 38105, USA. |
| Paul V. Motika | Comprehensive Epilepsy Center, Oregon Health and Science University, Portland, OR 97239 USA. |
| Saul A. Mullen | Epilepsy Research Centre, Department of Medicine, University of Melbourne (Austin Health), Heidelberg, Victoria 3084, Australia |
| Edward J. Novotny | Departments of Neurology and Pediatrics, University of Washington School of Medicine, Seattle Children’s Hospital, Seattle, Washington 98105 USA. |
| Terence J. O'Brien | Department of Neuroscience, Central Clinical School, Alfred Health, Monash University, Melbourne, Victoria, AUSTRALIA. |
| Karen L. Oliver | Epilepsy Research Centre, Department of Medicine (Austin Health), The University of Melbourne, Heidelberg, VIC 3031, Australia |
| Ruth Ottman | G. H. Sergievsky Center, Columbia University, New York, NY 10032, USA; and Department of Neurology, Columbia University, New York, NY 10032, USA; and Department of Epidemiology, Columbia University; and Division of Translational Epidemiology and Mental Health Equity, New York State Psychiatric Institute, New York, NY 10032 |
| Juliann M. Paolicchi | Department of Neurology and Pediatrics, Zucker Hofstra School of Medicine, SIUH and Lenox-Hill Hospital, New York, NY 10075 |
| Kristen L. Park | University of Colorado School of Medicine, Aurora CO 80045 USA |
| Sarah J. Paterson | Department of Paediatrics and Child Health, University of Otago, Wellington, New Zealand. |
| Slave Petrovski | Epilepsy Research Centre, Department of Medicine, University of Melbourne (Austin Health), Heidelberg, Victoria 3084, Australia. |
| William O. Pickrell | Wales Epilepsy Research Network, Swansea University Medical School, Swansea University, Wales, UK; and Neurology Department, Morriston Hospital, Swansea Bay University Health Board, Wales, UK. |
| Annapurna Poduri | Division of Epilepsy and Clinical Neurophysiology, Department of Neurology, Boston Children’s Hospital, Boston, Massachusetts 02115, USA. |
| Mark I. Rees | Faculty of Medicine & Health, University of Sydney, Sydney, Australia; Faculty of Medicine and Life Science, Swansea University, Swansea, Wales, UK |
| Lynette G. Sadleir | Department of Paediatrics and Child Health, University of Otago, Wellington, New Zealand. |
| Ingrid E. Scheffer | Epilepsy Research Centre, Department of Medicine, University of Melbourne (Austin Health), Heidelberg, Victoria 3084, Australia; and Florey Institute and Department of Paediatrics, Royal Children’s Hospital, Melbourne, Victoria, Australia. |
| Renée A. Shellhaas | Department of Neurology, Washington University School of Medicine, St. Louis, Missouri 63110 USA. |
| Elliott H. Sherr | Weill Institute of Neuroscience, Departments of Neurology, Pediatrics and Institute of Human Genetics, University of California, San Francisco, San Francisco, California 94158 USA. |
| Jerry J. Shih | Comprehensive Epilepsy Center, University of California San Diego, School of Medicine, La Jolla, CA, USA |
| Shlomo Shinnar | Albert Einstein College of Medicine, Bronx, NY. |
| Rani K Singh | Atrium health-Levine Children's Hospital, USA. |
| Joseph Sirven | Department of Neurology, Mayo Clinic, Scottsdale, Arizona 85259 USA. |
| Michael C. Smith | Rush University Medical Center, Rush Epilepsy Center, Chicago, IL. |
| Philip E.M. Smith | Department of Neurology, University Hospital of Wales, Heath Park, Cardiff, Wales, CF14 4XW, UK. |
| Michael R. Sperling | Jefferson Comprehensive Epilepsy Center, Department of Neurology, Sidney Kimmel Medical College at Thomas Jefferson University |
| Joseph Sullivan | Department of Neurology, University of California, San Francisco, San Francisco, California 94143 USA |
| Liu Lin Thio | Department of Neurology, Washington University School of Medicine, St. Louis, Missouri 63110 USA. |
| Rhys H. Thomas | Translational and Clinical Research Institute, Faculty of Medical Sciences, Newcastle University, UK. |
| Anu Venkat | Department of Pediatrics, Children’s Hospital at Saint Peter’s University Hospital, Rutgers Robert Wood Johnson Medical School, New Brunswick, NJ, USA. |
| Eileen P. G. Vining | Department of Neurology, Johns Hopkins Hospital, Baltimore, Maryland 21287 USA. |
| Gretchen K. Von Allmen | Division of Child Neurology, Department of Pediatrics, McGovern Medical School, University of Texas at Houston, Houston, TX 77030. |
| Judith Weisenberg | Department of Neurology, Washington University School of Medicine, St. Louis, Missouri 63110 USA. |
| Peter Widdess-Walsh | Department of Neurology, Beaumont Hospital, Dublin, Ireland. |
| Melodie R. Winawer | G. H. Sergievsky Center, Columbia University, New York, NY 10032, USA; and Department of Neurology, Columbia University, New York, NY 10032, USA. |
