# Supplementary material for: Phenotypic heterogeneity in familial epilepsies is influenced by polygenic risk for generalized and focal epilepsies
Source: Epilepsia. 2025 Mar 6;66(6):2036–45. doi: 10.1111/epi.18348 (PMC12169383; doi:10.1111/epi.18348)
Supplement: Supplementary file 2 — Figure S1. [file EPI-66-2036-s001.docx]

**SUPPORTING INFORMATION**

**Polygenic risk for generalized and focal epilepsies influence heterogeneity between and within familial epilepsies**

Colin A. Ellis, Ruth Ottman, Michael P. Epstein, Epi4K Consortium, Samuel F. Berkovic, Karen L. Oliver

Table of Contents

[Supplemental Methods 2](#_Toc188385991)

[Supplemental Figure 1. Ancestry principal components 3](#_Toc188385992)

[Supplemental Figure 2. Variance explained by principal components 4](#_Toc188385993)

[Supplemental Figure 3. Sensitivity analysis removing unclassified individuals 5](#_Toc188385994)

[Supplemental Figure 4. Negative control experiments 6](#_Toc188385995)

[Supplemental Figure 5. Senstivity analysis of p-value thresholds for PRS model construction 7](#_Toc188385996)

[Supplemental Figure 6. Heterogeneity within mixed families: pairwise analysis 8](#_Toc188385997)

[Supplemental Table 1. Pairwise analysis, statistical results 8](#_Toc188385998)

[Supplemental Table 2. Polygenic risk scores for generalized and focal epilepsy as predictors of individual phenotypes within mixed families 9](#_Toc188385999)

# Supplemental Methods

***Pairwise analysis of phenotypic heterogeneity within families***

We measured the pairwise difference in PRS between pairs of relatives within the mixed families. Whereas the mixed effects models adjust for relatedness as a covariate, this approach directly compares individuals only to their own family members. A single individual could contribute to multiple pairs. For GGE_PRS we calculated the score of the individual with generalized epilepsy minus the score of each of their relatives with focal epilepsy. For Focal_PRS we calculated the score of the individual with focal epilepsy minus the score of each of their relatives with generalized epilepsy. In both cases the difference scores would be hypothesized to be greater than zero. As a control group we used concordant pairs of relatives with the same phenotypes (both generalized, or both focal). Because the ordering of relatives within concordant pairs is arbitrary, we ran 1000 permutations randomly re-ordering the concordant pairs to derive the null distribution mean (expected value of zero) and variance for confidence intervals. For each iteration, we compared discordant pairs to concordant pairs using linear mixed effects models with PRS difference score as outcome, pair type (discordant vs concordant) as predictor of interest, kinship as fixed effect covariate, and family identifier as random effect covariate. We report the mean coefficients, standard errors, and p-values across the 1000 iterations of those models.

# Supplemental Figure 1. Ancestry principal components

The study cohort of cases and controls were merged with 1000 genomes and principal components (PCs) calculated to assess population stratification. Cases and controls for this project were restricted to samples that fell within +/- 4 standard deviations of the 1000 genomes European population mean for PC1 and PC2.


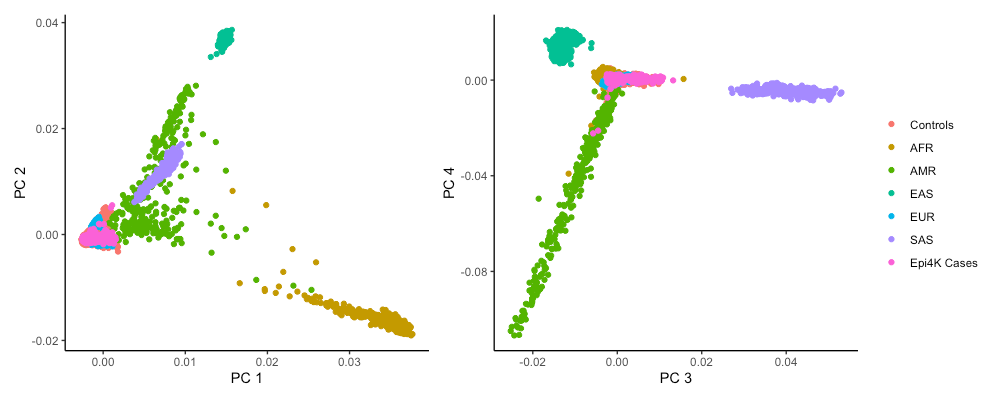


# Supplemental Figure 2. Variance explained by principal components

The first four principal components collectively explained 95% of the total genetic variance in the study cohort, and were used as covariates in our analyses.


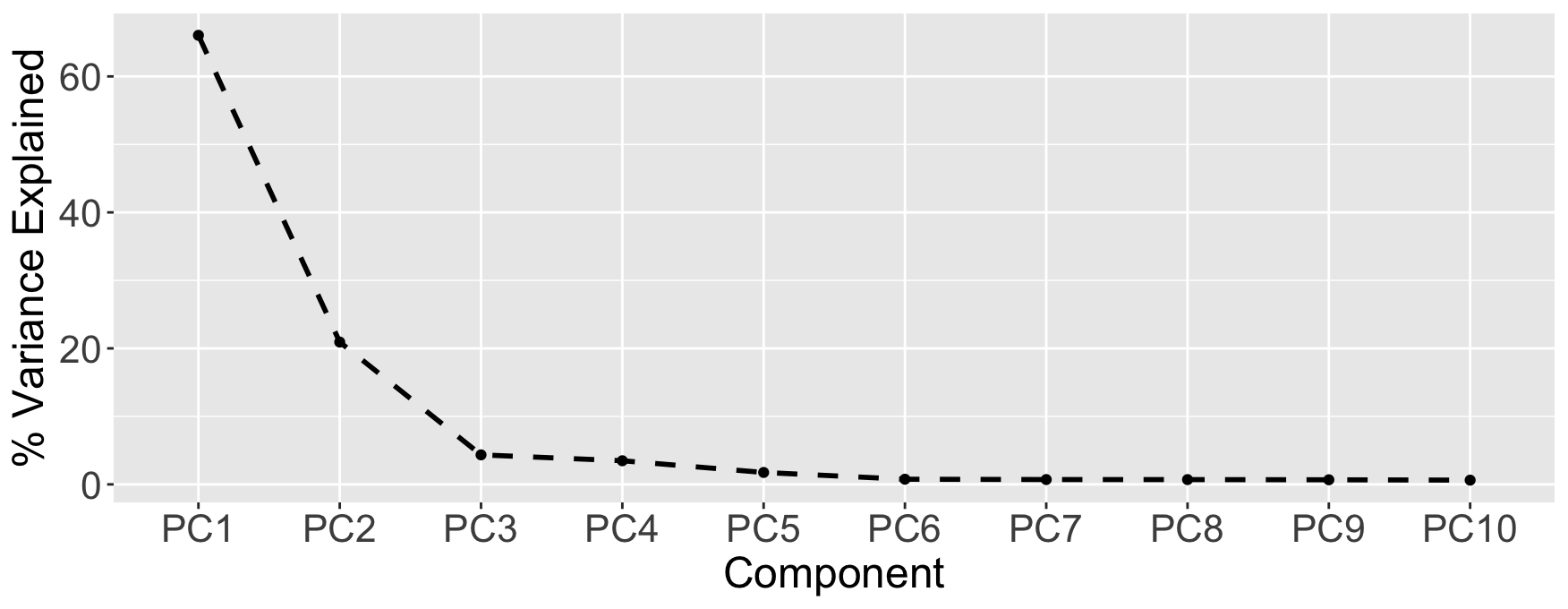


# Supplemental Figure 3. Sensitivity analysis removing unclassified individuals

The primary analysis included individuals with unclassified epilepsy (n=94). The sensitivity analysis removed these individuals and otherwise repeated the primary analysis.


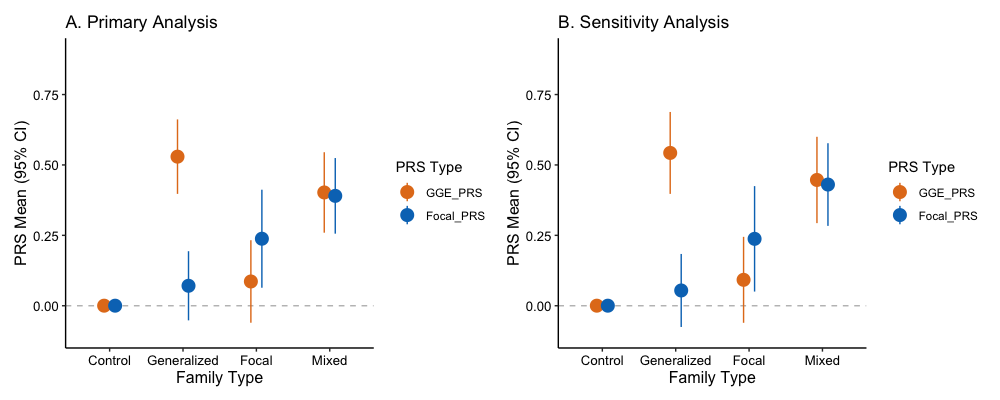


# Supplemental Figure 4. Negative control experiments

In negative control experiments, all three family types had PRS scores similar to controls for the non-neurologic conditions asthma and inflammatory bowel disease.


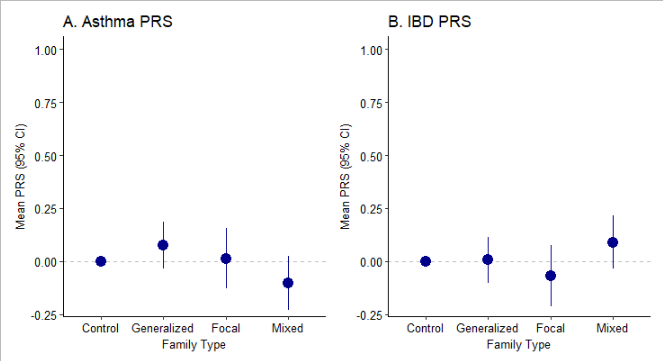


# Supplemental Figure 5. Sensitivity analysis of p-value thresholds for PRS model construction

Shown are the mean (95% CI) PRS for GGE epilepsy (orange/panel A) and focal epilepsy (blue/panel B) for members of mixed families who have generalized epilepsy (n = 100) or focal epilepsy (n = 84). The primary analysis used a p-value threshold of 0.5. Repeating the analysis using more stringent p-value thresholds for selecting the variants included in the PRS models eliminated the significant difference of GGE_PRS between individuals with generalized and individuals with focal epilepsy, and did not change the non-significant difference of PRS_Focal between the different phenotypes.


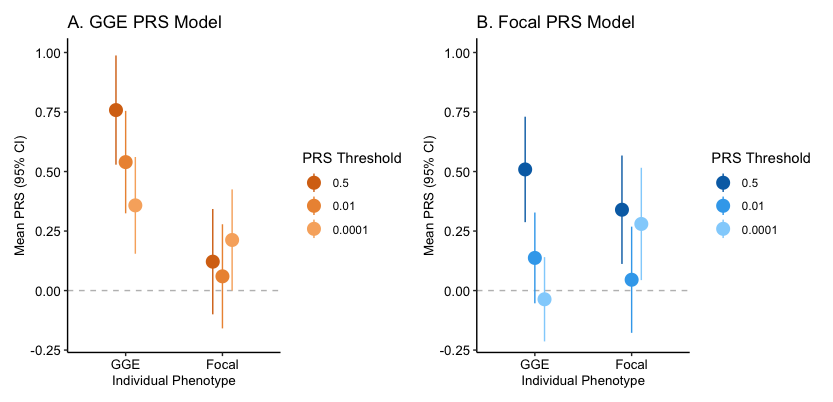


# Supplemental Figure 6. Heterogeneity within mixed families: pairwise analysis

Shown are the pairwise PRS difference between relatives within mixed families. Concordant pairs (n = 89 pairs) have the same phenotype and served as the control group. Discordant pairs (n = 86 pairs) have different phenotypes. (A) GGE_PRS was higher in individuals with a GGE phenotype than their relatives with a focal epilepsy phenotype, indicated by a mean pairwise PRS difference score greater than zero that was higher than concordant pairs and this difference was significant. (B) In contrast, Focal_PRS was not different in individuals with a focal epilepsy phenotype compared to their relatives with generalized epilepsy. Statistical testing results are presented in Supplemental Table 1 below.


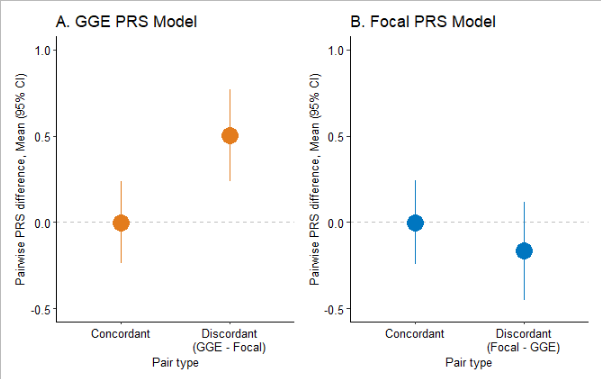


# Supplemental Table 1. Pairwise analysis, statistical results

|  | Pairwise PRS difference score, mean (95% CI) | | Mixed effects model results | |
| --- | --- | --- | --- | --- |
| PRS Model | Concordant pairs | Discordant pairs | Coefficient (SE) | p-value |
| (A) GGE_PRS | 0.00 (-0.24, 0.24) | 0.50 (0.24, 0.77) | 0.46 (0.18) | 0.03 |
| (B) Focal_PRS | 0.00 (-0.24, 0.24) | -0.17 (-0.45, 0.12) | -0.10 (0.18) | 0.51 |

# Supplemental Table 2. Polygenic risk scores for generalized and focal epilepsy as predictors of individual phenotypes within mixed families

We measured the extent to which PRS for generalized and focal epilepsies were predictive of an individual’s phenotype within these mixed families. For each analysis, the outcome was individual phenotype (focal versus generalized). Shown in the tables are the results of logistic mixed effects logistic regression model using phenotype (generalized or focal) as outcome variable; GGE_PRS, Focal_PRS, and their interaction as fixed effects; and family identifier as random effect. The full model (D) contained all the fixed effects predictors. Model (A) contained only the GGE_PRS predictor. Model (B) contained only the Focal_PRS predictor. Model (C) contained both PRS predictors, but not their interaction term.

A. GGE_PRS only

| **Predictor** | **Coefficient** | **SE** | **OR (95% CI)** | **p-value** |
| --- | --- | --- | --- | --- |
| GGE_PRS | 0.53 | 0.15 | 1.70 (1.28, 2.26) | 3 x 10^-4^ |

Tjur’s pseudo-R^2^ = 0.100

B. Focal_PRS only

| **Predictor** | **Coefficient** | **SE** | **OR (95% CI)** | **p-value** |
| --- | --- | --- | --- | --- |
| Focal_PRS | 0.15 | 0.14 | 1.16 (0.88, 1.52) | 0.29 |

Tjur’s pseudo-R^2^ = 0.008

C. GGE_PRS and Focal_PRS

| **Predictor** | **Coefficient** | **SE** | **OR (95% CI)** | **p-value** |
| --- | --- | --- | --- | --- |
| GGE_PRS | 0.59 | 0.16 | 1.80 (1.30, 2.48) | 3 x 10^-4^ |
| Focal_PRS | -0.12 | 0.16 | 0.88 (0.65, 1.21) | 0.44 |

Tjur’s pseudo-R^2^ = 0.104

D. GGE_PRS, Focal_PRS, and their interaction

| **Predictor** | **Coefficient** | **SE** | **OR (95% CI)** | **p-value** |
| --- | --- | --- | --- | --- |
| GGE_PRS | 0.57 | 0.16 | 1.77 (1.28, 2.45) | 5 x 10^-4^ |
| Focal_PRS | -0.17 | 0.18 | 0.84 (0.59, 1.19) | 0.33 |
| GGE_PRS x Focal_PRS | 0.08 | 0.12 | 1.08 ( 0.85, 1.38) | 0.52 |

Tjur’s pseudo-R^2^ = 0.108
